# Pharma[e]cology: How the Gut Microbiome Contributes to Variations in Drug Response

**DOI:** 10.1146/annurev-pharmtox-022724-100847

**Published:** 2024-12-17

**Authors:** Kai R. Trepka, Christine A. Olson, Vaibhav Upadhyay, Chen Zhang, Peter J. Turnbaugh

**Affiliations:** 1 Department of Microbiology & Immunology, University of California, San Francisco, California, USA;; 2 Department of Medicine, University of California, San Francisco, California, USA; 3 Chan Zuckerberg Biohub San Francisco, San Francisco, California, USA

**Keywords:** human microbiome, pharmacomicrobiomics, pharmacokinetics, pharmacodynamics, metabolism, absorption

## Abstract

Drugs represent our first, and sometimes last, line of defense for many diseases, yet despite decades of research we still do not fully understand why a given drug works in one patient and fails in the next. The human gut microbiome is one of the missing puzzle pieces, due to its ability to parallel and extend host pathways for drug metabolism, along with more complex host–microbiome interactions. Herein, we focus on the well-established links between the gut microbiome and drugs for heart disease and cancer, plus emerging data on neurological disease. We highlight the interdisciplinary methods that are available and how they can be used to address major remaining knowledge gaps, including the consequences of microbial drug metabolism for treatment outcomes. Continued progress in this area promises fundamental biological insights into humans and their associated microbial communities and strategies for leveraging the microbiome to improve the practice of medicine.

## INTRODUCTION

The human gastrointestinal (GI) tract is home to the gut microbiota, a complex community composed of microscopic organisms from all three domains of life: bacteria, archaea, and eukaryotes. These microorganisms encode millions of unique genes, collectively termed the gut microbiome, that extend the metabolic potential encoded by the human genome (1, 2). While the pathways through which gut microbiota metabolize endogenous (3) and diet-derived (4) compounds have been the subject of intensive study for decades, far less is known about the mechanisms through which the gut microbiome contributes to the metabolism of xenobiotics (compounds foreign to the human body, including antibiotics and host-targeted drugs) (5). Furthermore, emerging data suggest that the gut microbiome has broader impacts on pharmacokinetics than previously appreciated from studies of drug metabolism, altering all four components of drug ADME: absorption, distribution, metabolism, and elimination (6, 7). These observations, combined with the growing literature on host–microbiome interactions relevant to host metabolism and immunity, open up the potential for microbiome-dependent pathways that influence both pharmacokinetics and pharmacodynamics across a wide range of disease areas (8, 9).

Translation of these fundamental discoveries into the drug development pipeline and medical practice has lagged behind basic research linking the microbiome to ADME. Pharmaceutical industry standards for drug metabolism and pharmacokinetics rarely account for the impact of the microbiota on drug ADME properties (10). Standardized methods for in vitro testing of lead drug candidates for microbial metabolism remain lacking and pharmacokinetics studies of preclinical animal models rarely assess the potential role of interindividual or interspecies differences in the microbiome (10). This oversight has potentially far-reaching implications; however, it is understandable given the major remaining gaps in scientific knowledge coupled with the need for specialized and interdisciplinary expertise in the conceptual and methodological approaches necessary to work at the interface between microbiome and pharmacology research.

Many key gaps in our understanding arise from limitations in the methodologies used to study the role of the microbiome in pharmacology. Most current studies of microbial drug metabolism use culture-dependent methods, either by growing bacterial isolates in the presence of drugs (11, 12) or through the ex vivo incubation of human stool samples (13). While these methods have many practical advantages, they cannot be used to interrogate members of the gut microbiota that are difficult to culture and are limited in their ability to model the role of environmental factors like diet and host metabolites or more indirect mechanisms through which the microbiome alters drug disposition and mechanism of action. Furthermore, identifying the enzymes responsible and the resulting metabolites remains a major bottleneck (13).

In this review, we focus on two disease areas in which extensive and rigorous literature has linked the human gut microbiome to treatment outcomes in humans and preclinical models: cardiovascular disease and cancer. We also review emerging data relating to the treatment of neurological disease. We discuss the current state of insight into the mechanisms through which the human gut microbiota affects drugs that remain essential components of current medical practice. Furthermore, we highlight the remaining major knowledge gaps and opportunities for future studies at the intersection of pharmacology and microbiome research, including the need to develop computational and experimental strategies to accelerate the discovery of the most clinically relevant microbial species and enzymes capable of carrying out drug biotransformations. These efforts will be complemented by microbiome-directed randomized control trials in human subjects and paired studies of mouse models of disease assessing the physiological relevance of specific microbiome components for drug efficacy and side effect profiles.

## TAKING DRUG–MICROBIOME INTERACTIONS TO HEART

**Figure 1:**
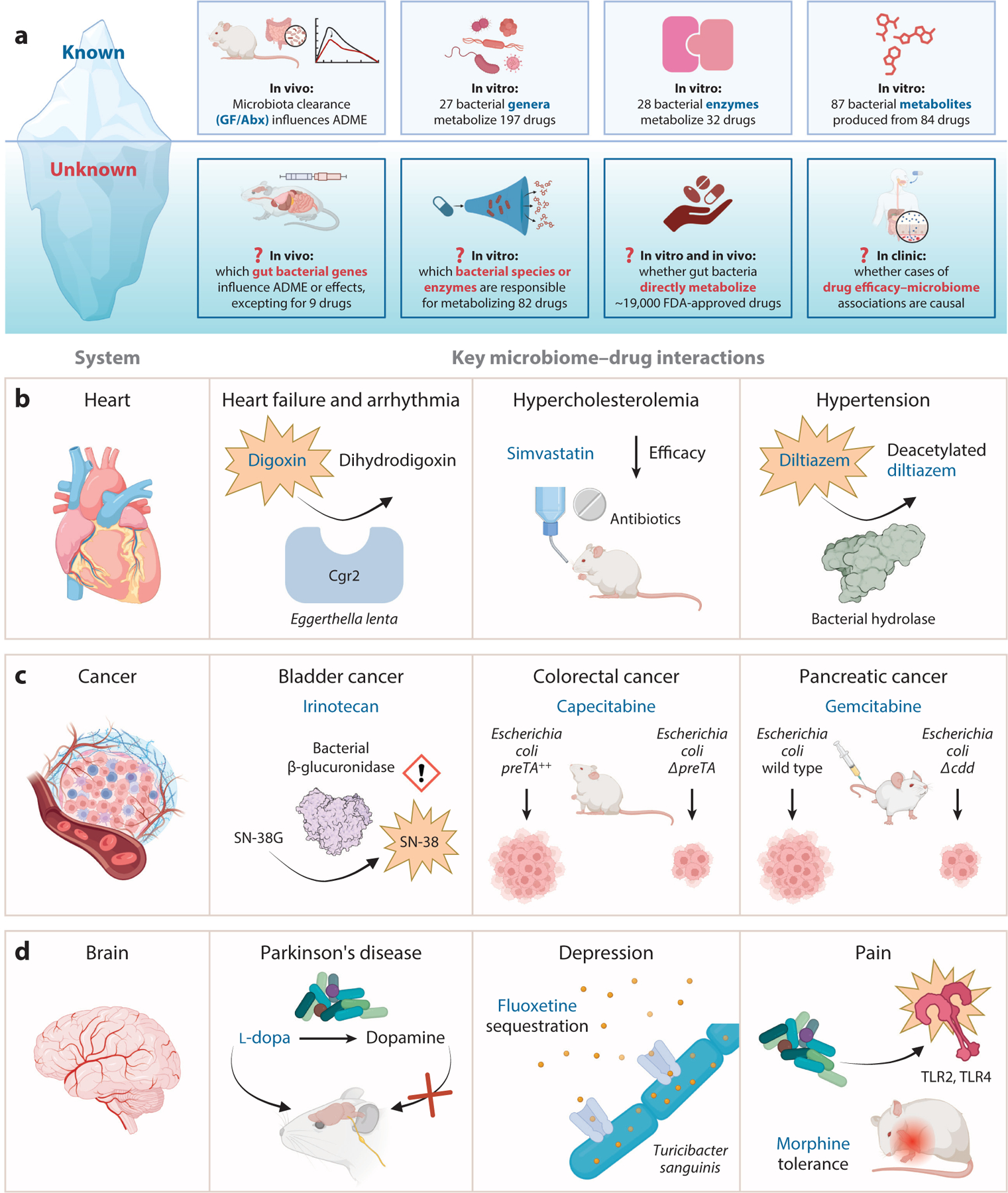
The gut microbiome affects drugs used to target multiple diseases. (*a*) It is now clear that our current understanding is just at the beginning stages, opening up many questions about the mechanisms through which the gut microbiome affects drug metabolism and disposition and their physiological/clinical relevance. (*b–d*) Representative examples of the impact of the gut microbiome on drugs used to treat (*b*) heart conditions, (*c*) cancer, and (*d*) the brain. (*b*) Digoxin, a treatment for heart failure and arrhythmia, is metabolized by *Eggerthella lenta* Cgr2 to dihydrodigoxin. Simvastatin, used to lower hypercholesterolemia, exhibits reduced efficacy in mouse models treated with antibiotics. Bacterial hydrolases deacetylate diltiazem, an antihypertensive drug. (*c*) Bacterial β-glucuronidase converts the inactive irinotecan metabolite SN-38G into active SN-38, causing increased toxicity. Capecitabine is more effective against tumor xenografts in mice colonized with *Escherichia coli ΔpreTA* than in mice colonized with *E. coli* overexpressing *preTA* (*E. coli preTA*^++^). Gemcitabine, a chemotherapeutic agent, decreased tumor sizes for mice colonized with *E. coli Δcdd* but not for mice colonized with wild-type *E. coli*. (*d*) Both host and gut bacterial enzymes decarboxylate the Parkinson’s disease treatment l-dopa to dopamine, which cannot cross the blood-brain barrier to exert therapeutic benefit. Fluoxetine, a selective serotonin reuptake inhibitor used to treat depression, is sequestered by the gut bacterium *Turicibacter sanguinis*. Gut bacterial colonization also activates TLR2 and TLR4, increasing morphine tolerance. Abbreviations: ADME, absorption, distribution, metabolism, and elimination; Cgr2, cardiac glycoside reductase 2; FDA, US Food and Drug Administration; GF, germ-free; l-dopa, levodopa; TLR2/4, Toll-like receptor 2/4. Figure adapted from images created with BioRender.com.

A textbook example of gut bacterial drug metabolism comes from digoxin (14), a cardiac glycoside drug used to treat heart failure and arrhythmias whose bioavailability is increased by broad-spectrum antibiotics due to limiting gut microbial metabolism (15, 16). However, recent studies indicate that the impact of the gut microbiota extends beyond digoxin to many other essential medications for heart disease (Figure 1*a*,*b*; Supplemental Table 1). Furthermore, mechanistic insights into the metabolism of digoxin and other drugs have led to surprising results that are relevant to the pathogenesis of other diseases. Herein, we highlight recent progress in this area and opportunities for future study.

Digoxin is reduced by select strains of *Eggerthella lenta* to the inactive metabolite dihydrodigoxin (17). A combination of comparative genomics and transcriptomics identified a two-gene operon that is highly induced by digoxin and found only in a subset of *E. lenta* strains, termed the cardiac glycoside reductase (*cgr*) operon (17). Both genes (*cgr1* and *cgr2*) encode predicted reductase enzymes; the purified Cgr2 protein is sufficient to catalyze the reduction of digoxin (18). This enzymatic activity is restricted to other closely related compounds within the cardenolide group of steroids (18), prompting questions about how these genes evolved given that cardenolides are highly toxic to humans and not a routine part of the mammalian diet.

Surprisingly, Cgr2 has endogenous substrates that affect intestinal immune activation (19, 20). Colonization of germ-free (GF) mice and conventionally raised (CONV-R) mice with *E. lenta* DSM2243 significantly increased T helper 17 (Th17) cells within the distal small intestine (ileum) and colon. Consistent with the established pathologic role of Th17 cells in numerous autoimmune diseases (21), *E. lenta* DSM2243 increased severity of colitis in the dextran sulfate sodium and IL-10 knockout mouse models. Comparisons of *E. lenta* strains in an in vitro T cell skewing assay and in mice revealed that Th17 cell activation is restricted to strains that carry the *cgr* operon (19). Preliminary attempts at identifying the Cgr2 substrate led to the discovery of two putative steroidal glycosides; however, more definitive purification and structural elucidation of the endogenous substrate(s) of Cgr2 remain outstanding. As expected, colonization of GF mice with a genetically engineered *Δcgr* strain led to significantly decreased colonic Th17 cells relative to wild-type controls (20). In contrast, ileal Th17 cells remained high (20), suggesting that *E. lenta* may have *cgr*-independent effects on the immune system that could be more broadly relevant to the treatment of diseases outside the cardiovascular system.

**Figure 2:**
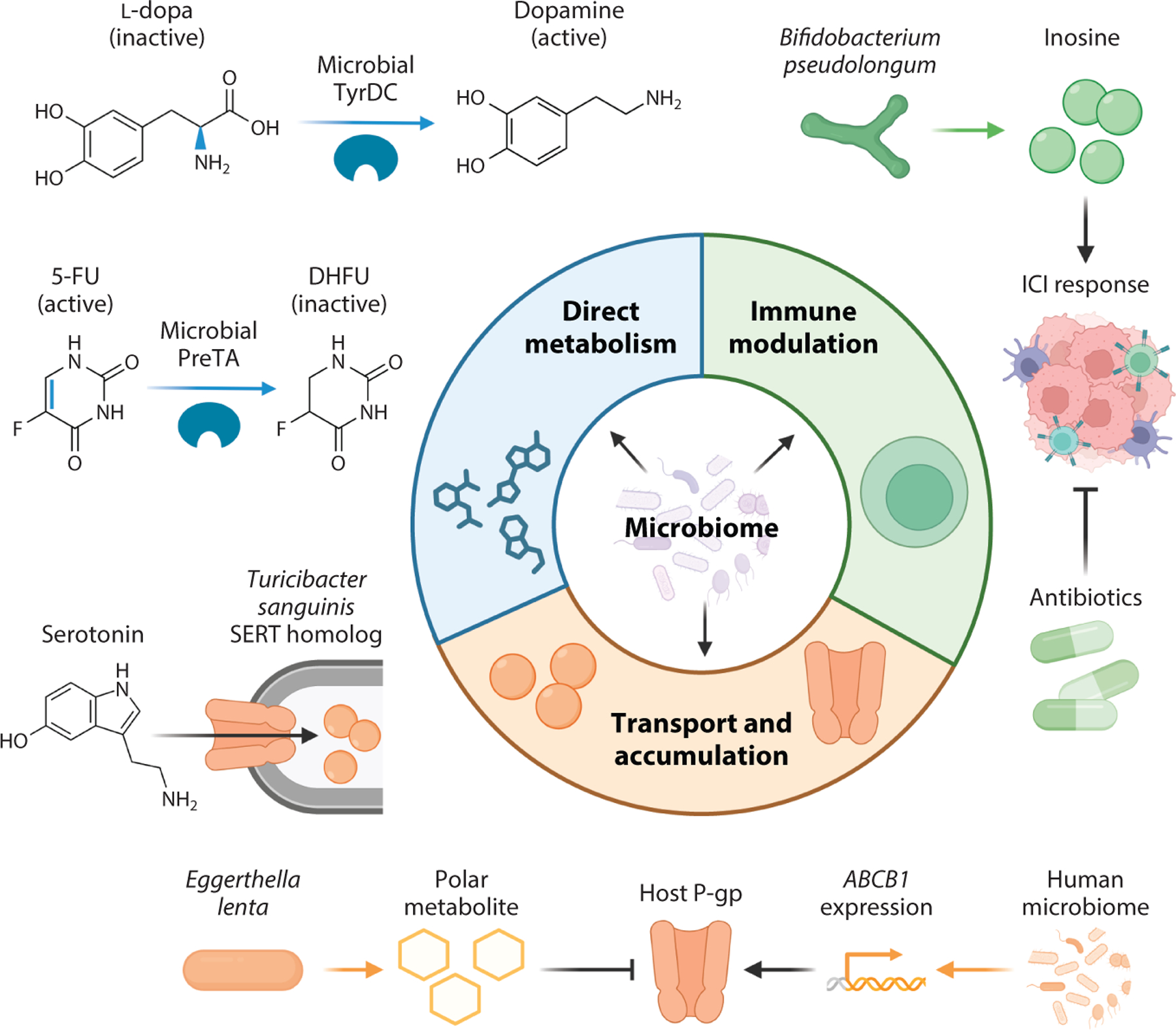
Hallmarks of pharmacomicrobiomics. Members of the gut microbiota influence the absorption, distribution, metabolism, and elimination of drugs through at least three key mechanisms: direct metabolism, transport and accumulation, and immune interactions. Gut microbes can directly metabolize drugs, prematurely activating prodrugs such as l-dopa or inactivating drugs such as 5-FU. Gut bacteria can modulate how the immune system interacts with immunomodulatory drugs, including the alteration of ICI efficacy through bacterial metabolites and small molecules. Finally, gut bacteria can alter drug transport, either through altering expression and activity of host drug transporters such as P-gp or by sequestering the drug through bacterial homologs of host drug transporters such as SERT. Abbreviations: 5-FU, 5-fluorouracil; DHFU, dihydrofluorouracil; ICI, immune checkpoint inhibitor; l-dopa, levodopa; P-gp, P-glycoprotein; SERT, serotonin transporter. Figure adapted from images created with BioRender.com.

Work in mouse models has also begun to reveal *cgr*-independent effects of *E. lenta* on digoxin pharmacokinetics (22). Colonization of GF mice with *E. lenta* DSM2243 led to a significant increase in peak serum digoxin levels, suggesting an effect of this bacterium on drug absorption. Digoxin is a substrate for the multidrug efflux transporter P-glycoprotein (P-gp) (23). *E. lenta* DSM2243 produces soluble P-gp inhibitors that inhibit the ATP hydrolysis activity required for P-gp to function. This activity appears to be unique to *E. lenta* and related members of the Eggerthellaceae family (22). The identification of the biosynthetic pathway and metabolites responsible will provide new opportunities to assess the trade-offs between gut bacterial drug metabolism and absorption. Furthermore, gut bacteria can also upregulate the intestinal expression of P-gp (22, 24, 25), suggesting that the activity of this transporter may reflect a balance between microbiome-dependent activation and inhibition (Figure 2).

The complexity of drug–microbiome interactions is now becoming more apparent for statins, including atorvastatin and simvastatin, the third and fifth most prescribed drugs in the United States, respectively (26). Statins are used to decrease low-density lipoprotein cholesterol in the blood, which is a risk factor for atherosclerosis (27). Their primary mechanism of action is through inhibiting 3-hydroxy-3-methylglutaryl coenzyme A (HMG-CoA) reductase, the rate-limiting step for cholesterol biosynthesis in the mevalonate pathway (27). Surprisingly, the growth of diverse human gut bacteria is inhibited by statins (28, 29), even though bacteria encode an alternative pathway for isoprenoid biosynthesis, the methylerythritol phosphate pathway, which does not require HMG-CoA reductase (30, 31). In turn, statins can be metabolized by gut microbial monocultures and ex vivo stool communities (11, 13, 32, 33).

While the direct target of statins in human-associated bacterial cells remains unclear, multiple mechanisms can enable gut bacteria to resist their antimicrobial effects (28). Transcriptomics analysis demonstrated that simvastatin induces various genetic loci regulated by the multiple antibiotic resistance regulator (MarR) in the gut Actinobacterium *E. lenta*, including a gene cluster predicted to affect fatty acid biogenesis. Paired transcriptomics and transposon sequencing in *Bacteroides thetaiotaomicron* led to the identification of three distinct AcrAB-TolC efflux systems that are induced by simvastatin and protect against its growth inhibitory effects. The copy number of AcrAB-TolC efflux systems varies substantially among phyla, with up to seven in a single genome of *Bacteroidota* versus a maximum of two in *Pseudomonadota*. Deletion of the single *tolC* in *Escherichia coli* BW25113 made a fully resistant strain sensitive to simvastatin (28), emphasizing the importance of genetic redundancy in protecting against the off-target effects of commonly used drugs. An independent study focused on high-throughput transcriptional profiling of hundreds of drug–bacteria pairs highlighted the reproducible ability of simvastatin to induce the expression of AcrAB-TolC efflux systems across multiple bacterial strains (34). In contrast, atorvastatin had a more selective effect, inducing AcrAB-TolC only in *Parabacteroides distasonis* (34), providing some clues about the structural motifs that may be necessary for detection.

Multiple observational studies of humans (35–38) have identified associations between statin treatment outcomes and the gut microbiota, supporting the translational relevance of this area of inquiry. Several gut bacterial bile acid metabolites are associated with statin efficacy, potentially due to altered intestinal absorption of statins (37). More recently, an analysis of 16S rRNA gene sequencing (16S-seq) data from 1,512 subjects revealed associations between the gut microbiota and statin response (35). However, it remains difficult to identify associations that are consistent across cohorts (35, 36), potentially due to the confounding effects of differences in host pathophysiology, diet, or ethnicity (38). Mechanistic insights into the various pathways through which the microbiome affects statin response are essential to help design follow-up observational or interventional human studies. Notably, antibiotic treatment interferes with the efficacy of simvastatin in mice (39), providing a tractable model for future studies.

The calcium channel blockers amlodipine (40) and diltiazem (11), which are used to treat hypertension, are also subject to gut bacterial metabolism, potentially interfering with their effects. In a high-throughput screen of 271 orally administered drugs, the model gut bacterium *B. thetaiotaomicron* decreased the levels of 46 drugs, including diltiazem (11). Heterologous expression of *B. thetaiotaomicron* genes in *E. coli* implicated the *bt4096* gene locus in diltiazem metabolism, which was validated in GF mice mono-associated with wild-type and *Δbt4096 B. thetaiotaomicron* strains by quantifying the predicted microbial metabolite: deacetylated diltiazem (11). The crystal structure of BT4096 revealed similarities to a carbohydrate-degrading acetylesterase, suggesting that this enzyme may have evolved to target acetylated sugars while having an open binding pocket to allow diltiazem to enter the active site (41). Similarly, incubation of cell lysates from human stool samples led to a decrease in amlodipine levels and a corresponding increase in an inactive pyridine metabolite (40). Consistent with these results, ampicillin increased the oral bioavailability of amlodipine in rats and decreased the enzymatic activity found in stool samples (40). This picture was recently made more complex with the discovery that the interaction between amlodipine and the microbiome is bidirectional. Amlodipine itself is broadly toxic to distantly related gut isolates and induces the expression of multidrug resistance pathways associated with efflux transporters (34).

The impact of the microbiome also extends beyond therapeutics to recreational drugs that increase the risk of cardiovascular and other diseases. Nicotine can accumulate in the distal small intestine (ileum) of cigarette smokers and is higher in GF mice than in CONV-R mice (42). Paired sequence analysis and culture-based experiments led to the identification of a gene encoded by *Bacteroides xylanisolvens* termed *nicX*, which facilitates intestinal nicotine degradation and accumulation of the third-hand smoking compound 4-hydroxy-1-(3-pyridyl)-1-butanone (42). *B. xylanisolvens* reduces nicotine accumulation in CONV-R mice and a *ΔnicX* strain was unable to deplete nicotine during in vitro growth (42). Given the far-ranging health impacts of nicotine and its metabolites, these findings are potentially relevant to many disease areas, including heart disease (43); however, the initial study focused on the liver. This was motivated by the fact that nicotine activates intestinal AMP-activated protein kinase alpha, elevating the sphingolipid ceramide and promoting liver inflammation in mice. *B. xylanisolvens* is also negatively correlated with markers of inflammation in patients with nonalcoholic steatohepatitis (42).

Studies of microbial interactions with endogenous hormones may also provide important information about the role of the microbiome in pharmacology and vice versa. The hormones norepinephrine and epinephrine are used therapeutically to increase blood pressure (14). Norepinephrine is converted by *E. coli* into 3,4-dihydroxymandelic acid in a two-step process requiring aldehyde dehydrogenase (44). Both compounds are intended to target host G protein–coupled receptors that engage the fight-or-flight response in mammals (45); however, they can also interact with a bacterial two-component system important for quorum sensing (46). Norepinephrine also induces the expression of genes important for the pathogenicity of enterohemorrhagic *E. coli* (47, 48). GF mice have lower levels of the biologically active, unconjugated norepinephrine in the gut lumen, with a putative mechanism dependent on gut bacterial β-glucuronidase (49). More work is needed to study whether these off-target effects impact the therapeutic outcomes of these drugs and how these and other hormones interact with the broader set of strains and taxonomic groups found within the human microbiota.

## MICROBIAL ALLIES AND ADVERSARIES IN THE WAR ON CANCER

Despite advances in anticancer biologics and cell therapies, small-molecule drugs remain the cornerstone of cancer therapy for solid tumors (50). Anticancer drugs are often delivered as prodrugs and canonically thought to be sequentially activated and inactivated by host metabolism (51). However, microbial enzymes have the potential to affect many of these steps directly or indirectly, with consequences for overall drug disposition and tumor drug exposure (52) (Figure 1*c*). In this section, we review recent progress in this area and key gaps in our current knowledge. We also discuss the exciting and emerging evidence that the microbiome is also relevant to cutting-edge immunotherapies and even cell therapy.

Microbial reactivation of anticancer drugs can increase local active drug levels, leading to GI side effects. For example, irinotecan (CPT-11) is a first-line treatment for many solid tumors, with a dose-limiting toxicity of severe diarrhea (53). The prodrug CPT-11 is activated by nonspecific esterases into active SN-38 (7-ethyl-10-hydroxycamptothecin), which poisons DNA topoisomerase I, preventing DNA replication and cell proliferation (53). Canonically, hepatic glucuronosyltransferases convert SN-38 into SN-38G (SN-38 glucuronide), which is then excreted in the stool (53). However, β-glucuronidases expressed by diverse gut bacteria can reactivate SN-38G, increasing active drug levels in the gut and resulting in severe diarrhea (54). This toxicity can be rescued by inhibition of bacterial β-glucuronidase (54). Inhibition of bacterial β-glucuronidase does not alter the serum pharmacokinetics of SN-38 (55), suggesting that this transformation is more relevant for GI drug concentrations. CPT-11 is often administered with additional drugs to manage pain or other side effects. Morphine, an opioid used for pain management, leads to increased gut microbial β-glucuronidase activity, stool SN-38, and markers of small intestinal damage in mice (56).

Microbial nucleoside/nucleotide metabolism pathways can be repurposed for anticancer drug metabolism, with implications for host drug concentrations and resultant efficacy and toxicity. 5-Fluorouracil (5-FU) is a cytotoxic fluoropyrimidine antimetabolite used as a first-line treatment in many combination regimens. Host dihydropyrimidine dehydrogenase can convert active 5-FU into inactive dihydrofluorouracil (DHFU). However, a homologous bacterial enzyme is encoded by the *preTA* operon, including subunits PreT and PreA, which together carry out this transformation, resulting in altered 5-FU pharmacokinetics (57) (Figure 2). Gnotobiotic mice colonized with *preTA*-overexpressing *E. coli* have significantly lower maximum plasma concentrations of 5-FU relative to mice colonized with *ΔpreTA E. coli*. Remarkably, colonization of streptomycin-treated CONV-R mice with isogenic strains of *E. coli* differing in the level of *preTA* expression revealed that high levels of *preTA* can be sufficient to block the efficacy of oral capecitabine (a 5-FU prodrug) in a cancer xenograft model. Paired cross-sectional and longitudinal data in patients with cancer revealed that *preTA* is ubiquitous in the human gut microbiome but can vary by multiple orders of magnitude in abundance, potentially contributing to variations in treatment outcomes (57).

Similar to that in the host (58), the pathways for fluoropyrimidine drug metabolism in microbial cells are nutrient dependent. In *Caenorhabditis elegans*, vitamins B6 (pyridoxine) and B9 (folate) modulate the cytotoxic effect of 5-FU (59). 5-FU-metabolizing *E. coli* protects host worms from drug toxicity only when its biosynthetic pathways for vitamins B6 and B9 are genetically disrupted, an effect that is reversed by dietary supplementation of pyridoxine and folate (59). 5-FU-resistant strains of *E. coli* and other gut bacteria can be rapidly identified (57). Selection for drug resistance in *E. coli* under low-nutrient conditions (M9 minimal media) led to strains with a more marked impact on 5-FU toxicity in worms relative to strains selected in rich Luria broth media (60). Consumption of microorganisms in fermented foods and probiotic products may also affect 5-FU toxicity, potentially through altering the gut microbiome. *Lactobacillus* probiotics alleviated 5-FU-induced diarrhea in a mouse model (61) and a randomized clinical trial (62). These *Lactobacillus* strains encode *preTA* (57), potentially allowing them to directly affect intestinal drug metabolism.

The impact of bacteria on anticancer drugs extends beyond their role in intestinal metabolism and absorption. A growing literature suggests that some bacteria can proliferate within tumors in multiple body sites that are typically considered sterile (63). Specifically, tumor-resident *E. coli* can locally affect drug levels within the tumor microenvironment. *E. coli* isolated from patients with colorectal cancer tumors was cocultured with 5-FU and colorectal cancer cells, resulting in conversion of active 5-FU into inactive metabolite DHFU and higher cancer cell proliferation (64). *E. coli* encodes a cytidine deaminase (*cdd*) gene necessary to convert the anticancer thymidine mimic gemcitabine into 2′,2′-difluoro-2′-deoxyuridine (65). BALB/c mice subject to a subcutaneous colon carcinoma model were given tail vein injections of luciferase-labeled wild-type *E. coli* and *E. coli Δcdd* prior to treatment with gemcitabine (65). While both strains of *E. coli* localized to the tumor, gemcitabine prevented tumor growth only in mice treated with *E. coli Δcdd*, supporting the hypothesis that intratumoral bacterial *cdd* interferes with fluoropyrimidine efficacy in vivo (65). Gemcitabine can also select for multiple types of gemcitabine-resistant *E. coli* with opposing effects on cancer cell growth (66). Drug-resistant *E. coli* can overexpress *cdd*, decreasing active drug levels. On the other hand, drug-resistant *E. coli* can inactivate a key nucleoside permease (NupC), preventing bacterial drug uptake and resulting in higher drug exposure to cocultured cancer cells (66).

The scope of the microbiome’s role in cancer therapy extends far beyond drug metabolism, due to the intimate relationship between our associated microbial communities and the immune system. This is particularly important given the long history of small-molecule drugs delivered by infusion (which bypasses first-pass intestinal metabolism) and the more recent shift toward biologics and even cell-based therapies for cancer. Many of these drugs target the immune system, raising the potential for a microbiome-dependent pharmacodynamic effect.

This concept has been most well-studied in the context of immune checkpoint blockade. Immune checkpoint inhibitors (ICIs) prevent repressive interactions that inhibit T cell–mediated cancer killing, for example, by using antibodies to block cancer cell ligand PD-L1 from binding to PD-1 on CD8^+^ T cells (67). Mice purchased from different vendors have different melanoma growth and tumor-specific immune responses (68). Cohousing and microbiota transplantation experiments indicated that vendor-specific differences in the gut microbiota could be responsible (68). 16S-seq data associated *Bifidobacterium* sp. with improved αPD-L1 response, which was confirmed by administration of a cocktail of bifidobacterial strains (68). *Bacteroides* species have also been implicated in response to an alternative form of immunotherapy that relies on blocking CTLA-4 (69). MCA-205 sarcomas in GF mice did not respond to CTLA-4 blockade, whereas CONV-R controls had a clear response to treatment. A targeted screen in mice identified multiple *Bacteroides* strains that slow tumor growth, consistent with the immunostimulatory effects of *Bacteroides fragilis* (69).

Across multiple human cohorts, the gut microbiome is distinct between responders and nonresponders to checkpoint blockade (70–72). In patients with epithelial tumors treated with αPD-L1, recent antibiotic treatment was associated with a decrease in overall and progression-free survival (70). Similarly, in patients with metastatic melanoma treated with αPD-L1, increased microbial diversity was associated with improved survival (71). An independent analysis of the pretreatment stool microbiomes of patients with metastatic melanoma revealed an enrichment of multiple bacterial species in αPD-L1 responders, including *Bifidobacterium longum*, *Collinsella aerofaciens*, and *Enterococcus faecium* (72). Consistent differences in the microbiota across studies remain elusive (73), potentially due to the confounding effects of diet (74), ethnicity (75), or other medications (57). Furthermore, a recent report found that menaquinone-producing microbes were associated with decreased ICI-induced adverse events (76), consistent with a role for the microbiome in modulating toxicity as well as efficacy.

Support for a causal role of the human gut microbiome in immune checkpoint inhibition comes from microbiota transplantation experiments (70–72). MCA-205 sarcomas in GF mice colonized with the gut microbiota from patients who responded to αPD-L1 had an improved response relative to sarcomas in mice colonized by nonresponders, spanning multiple cancer types (70). Similar results were found in an independent study that used a different model of cancer (BRAF^V600E^/PTEN^−/−^melanoma) (71).

The mechanisms through which microorganisms affect ICIs are largely unknown. Cell wall components are important to consider, including the capsular polysaccharides produced by *B. fragilis* (69). Small molecules may also play a role (77). *Bifidobacterium pseudolongum* promotes an α-CTLA4 immunotherapy response in GF mice implanted with MC38 colon cancer cells (77). Untargeted metabolomics revealed that the purine nucleoside inosine was enriched in the sera of *B. pseudolongum* monocolonized mice (77). Inosine was sufficient to enhance immunotherapy response in GF mouse models of colorectal cancer, bladder cancer, and melanoma (77) (Figure 2).

Most recently, the treatment of cancer has been revolutionized by cell-based therapies. This pioneering approach engineers T cells that express a chimeric antigen receptor (CAR) with an extracellular single-chain fragment variable domain to bind tumor cell surface antigens and intracellular activation domains to enable tumor killing (78). While response rates for leukemia have been astonishing, reaching as high as 83% of patients with complete response (79), there are still major unmet needs to address the remaining nonresponders and to extend these approaches to solid tumors (80). Multiple studies have identified differences in the gut microbiota of CAR-T cell responders and nonresponders with leukemia, including enrichment of the *Faecalibacterium* and *Ruminococcus* genera in complete responders (81, 82). Antibiotics are associated with worsened CAR-T cell outcomes across multiple patient cohorts spanning leukemia, multiple myeloma, and B cell lymphoma (81–83); however, it remains unclear whether these associations are driven by comorbidities, infection control, disruption of the microbiota, or a combination thereof. Treatment of mice with oral vancomycin led to increased tumor control and antigen cross-presentation during CAR-T cell therapy, providing a tractable model for mechanistic dissection (84).

## MASTERING OUR MENTALLY MANIPULATIVE MICROORGANISMS

While the gut–brain axis has been a topic of intense study for many years, the primary focus to date has been on microbial risk factors of disease (85). The ability of the gut microbiome to influence drugs that target the central and peripheral nervous systems remains far less understood. Remarkably, gut bacteria can broadly deplete neurological drugs during in vitro growth (86) (Supplemental Table 1). However, the mechanisms responsible and their clinical impacts remain to be determined. Here, we highlight three key areas where mechanistic insights have already begun to emerge: Parkinson’s disease, depression, and pain (Figure 1*d*). These discoveries provide a proof of principle to assess the role of the gut microbiome more broadly in the treatment of neurological diseases.

The treatment of Parkinson’s disease has relied on levodopa (l-dopa) for more than 50 years (87). While l-dopa works well for many patients, nearly 50% of individuals develop multiple adverse effects, including dyskinesias (involuntary movements) and motor fluctuations (periods over the day where l-dopa is ineffective and symptoms recur) (88). l-dopa is sensitive to peripheral metabolism, motivating strategies to improve drug bioavailability and distribution while reducing side effects like nausea and dyskinesias (89). l-dopa is typically administered with drugs like carbidopa (α-methyldopahydrazine), which irreversibly binds pyridoxal 5′-phosphate, inhibiting the mammalian aromatic l–amino acid decarboxylase (AADC) (90). Yet carbidopa does not inhibit the metabolism of l-dopa by the gut microbiota (91, 92), providing a potential source of AADC-independent variations in drug metabolism and disposition.

Multiple human gut bacterial species have been implicated in the peripheral metabolism of l-dopa. *Enterococcus faecalis* can decarboxylate l-dopa to dopamine via the enzyme tyrosine decarboxylase (TyrDC) (91, 92) (Figure 2). The *tyrDC* gene is also found in other members of the *Enterococcus* genus and within *Lactobacillus* (91–93). Dopamine is further metabolized to *m*-tyramine by *E. lenta* dopamine dehydroxylase (Dadh) (91). The soil bacterium *Clostridium sporogenes* and multiple gut bifidobacterial species can also deaminate l-dopa (94, 95). Thus, it is likely the net effect of multiple distinct bacterial species and drug biotransformations that determines the extent of intestinal l-dopa metabolism prior to reaching general circulation. More work is needed to model the interactions between these different strains.

Multiple lines of evidence support the physiological and clinical relevance of gut bacterial *tyrDC* for l-dopa. Chemical inhibition of TyrDC with (*S*)-α-fluoromethyltyrosine significantly increased circulating levels of l-dopa in mice monocolonized with wild-type *E. faecalis* relative to vehicle controls (91). Data from patients with Parkinson’s disease revealed an association between *tyrDC* abundance and l-dopa efficacy (96). However, the relative importance of l-dopa decarboxylation versus deamination or other more indirect host–microbiome interactions relevant to disease remains to be explored.

In addition to its impact on dopamine levels, the gut microbiome has extensive interactions with serotonin [5-hydroxytryptamine (5-HT)]. Gut bacterial colonization stimulates the biosynthesis of 5-HT within the GI tract, leading to increased colonic and systemic 5-HT concentrations (97, 98). Surprisingly, gut bacteria can gain a direct energetic benefit from host 5-HT. *Turicibacter sanguinis* expresses a homolog of the mammalian serotonin transporter (SERT) that enables serotonin uptake (99) (Figure 2). In the treatment of depression, selective serotonin reuptake inhibitors (SSRIs) like fluoxetine inhibit SERT and increase synaptic serotonin. Treatment with fluoxetine induced *T. sanguinis* punctate membrane staining, a hallmark of sporulation, suggesting that lack of available 5-HT may trigger a dormant metabolic state (99). Pretreatment with fluoxetine impaired the ability of *T. sanguinis* to colonize the gut of antibiotic-treated mice (99). Fluoxetine treatment is also associated with decreased levels of *Turicibacter* in mice and human subjects (99, 100), providing initial support for the clinical relevance of the off-target effect of this medication. The broader relevance of gut bacteria for other SSRIs remains unclear; however, one study identified an association between the gut microbiome and patient responsiveness to the SSRI drugs citalopram and escitalopram, its (*S*)-enantiomer (101).

A growing body of literature has implicated the gut microbiome in the management of pain. Repeated use of morphine leads to decreased efficacy (i.e., tolerance) in CONV-R mice and patients (102). Antibiotic-depleted or GF mice gained a prolonged effect of morphine due in part to decreased activation of Toll-like receptors 2 and 4 (102, 103). The gut microbiota may also contribute to the side effects of drugs used for pain. Oral administration of aspirin altered the gut microbiota in human subjects and mice, with a consistent decrease in the relative abundance of *Parabacteroides goldsteinii* (104). Administration of *P. goldsteinii* to aspirin-treated mice rescued markers of GI toxicity, which was attributable to suppression of farnesoid X receptor signaling by the *P. goldsteinii* metabolite 7-keto-lithocholic acid (104).

In the case of acetaminophen, the gut microbiota has been implicated in drug toxicity outside of the GI tract. In the liver, excess acetaminophen can be converted by cytochrome P450 CYP2E1 into reactive chemical species, leading to oxidative damage and, consequently, acute liver failure (105). Oral administration of magnesium salt (Mg^2+^) rescued a mouse model of acetaminophen-induced liver toxicity in a microbiota-dependent manner, as evidenced by comparisons of CONV-R and GF mice, as well as microbiota transplantations from Mg-treated mice and human subjects relative to controls (105). This protective effect was associated with an Mg^2+^-dependent increase in the levels of gut bifidobacteria and their metabolite indole-3-carboxylic acid, which is sufficient to inhibit CYP2E1 activity (105).

## AN EXPANDING TOOL KIT FOR PHARMACOMICROBIOMICS RESEARCH

The first Gordon Research Conference on Drug Metabolism was held in 1971 and has been followed by more than 50 years of innovative research on this topic. Remarkably, the microbiome was a topic of discussion from the very beginning of this long-running conference series, featuring a talk by Robert R. Scheline, who had reviewed the considerable literature already available at that time (106). In parallel, the broader field of microbiome research had been highly productive, including seminal studies using culture-dependent methods and gnotobiotic mice that established many of the general areas that are studied today (107). Yet the ability to study these complex microbial ecosystems was limited by the tools available at the time. The advent of low-cost and higher-throughput sequencing methods enabled researchers to sequence the genomes of communities (metagenomics). Combined with improved methods for high-throughput culturing, gnotobiotic and transgenic mice, and metabolomics, researchers are now able to reduce these complex systems to their cellular and molecular mechanisms, providing unprecedented insight.

Given the rapidly expanding literature at the interface of the microbiome and pharmacology, we opted to focus this review primarily on heart disease and cancer because they are two well-developed areas of study, with neurological disease as an exciting and emerging topic. However, these are just the beginning (Figure 1*a*); the scope of the microbiome likely extends to most if not all disease areas, considering the potential for both direct and indirect effects on drug pharmacokinetics/pharmacodynamics (Figure 2). The examples we highlighted emphasize the benefits of complementary top-down and bottom-up approaches. Studies of the gut bacterial interactions with digoxin and statins began with clinical observations (15, 108), which have now been dissected in mechanistic detail in cell culture and animal models. In contrast, genetic and biochemical studies of enzymes relevant to anticancer drug metabolism (54, 57) led to the design of downstream experiments in mice and human subjects. This is especially important to consider now, given the rapid pace of discovery of high-throughput in vitro screens for microbial drug sensitivity and metabolism (11–13, 29), providing the ability to generate more data-driven hypotheses about the genes and enzymes responsible and the mechanisms that could be at play within the GI tract or other body habitats.

Identifying the genes and enzymes responsible for microbial drug metabolism remains a major bottleneck. For model organisms like *Bacteroides thetaiotaomicron*, it is possible to generate genome-wide transposon libraries (109), which can be screened for loss of the ability to metabolize a given drug (86). Alternatively, one can clone genomic DNA from genetically intractable bacteria into a model host organism, screening for gain of function (11). While powerful, both approaches have limitations, including the inability to study essential genes using transposon libraries and the requirement for small modular genetic elements compatible with heterologous expression. The former could potentially be addressed through transcriptional repression via CRISPR interference (110) and the latter would benefit from improved tools to construct and study large-insert libraries.

To address these experimental challenges, multiple computational tools have been developed that seek to predict genes responsible for biotransformations of interest. This includes the SIMMER tool (111), which stands for similarity algorithms that identify microbiome enzymatic reactions. SIMMER utilizes a chemical reaction vector-embedding approach for comparing metabolic reactions (112) combined with prior knowledge of microbial reactions in MetaCyc (113) and metagenomic data to assess gene prevalence. SIMMER identified known genes for 88% of a set of 33 known drug–metabolite pairs. As a test case, this algorithm was applied to study the metabolism of methotrexate, an antimetabolite drug used to treat rheumatoid arthritis and cancer. There was a significant overlap between computational predictions and experimental measurements of methotrexate depletion by human gut bacterial isolates. Furthermore, SIMMER-predicted genes for methotrexate metabolism were enriched in nonresponders to the drug, supporting the translational relevance of the gut microbiome for the treatment of rheumatoid arthritis (111, 114). Continued refinement of these and other computational tools promises to continue to accelerate progress, especially given the recent development of artificial intelligence–based tools for enzyme prediction (115).

More work is needed to computationally predict microbial metabolites, which can often be challenging to identify if the reactions are novel, if multiple metabolites are produced, or if the metabolites are volatile in nature. This is especially important given the common confounding effects of bioaccumulation (12), in which drug levels decrease due to binding to microbial cells, sequestration in the cytosol without metabolism, or both. Most drugs with in vitro evidence for microbial depletion have no identified metabolites (Supplemental Table 1), making the relative contributions of microbial bioaccumulation versus metabolism unknown.

Drugs can also be degraded due to abiotic or nonenzymatic mechanisms, which further complicates our ability to predict the effect of the microbiome on pharmacokinetics. This phenomenon has been recently studied in the context of drugs and food dyes that contain an azo (R–N D N–R′) bond (116, 117). Numerous gut bacteria produce hydrogen sulfide (H_2_S) as a metabolic end product, which can act as a redox partner with azo dyes (116). In *E. coli*, the well-studied azo reductase (*azoR*) gene is dispensable for azo dye depletion due to the redundant ability of H_2_S to degrade azo dyes (117). Dissection of the genes responsible revealed the importance of anaerobic conditions for enabling azo dye depletion through the fumarate and nitrate reduction (*fnr*) regulator and its downstream small noncoding regulatory RNA, *fnrS*. Furthermore, the production of H_2_S is dependent on the availability of l-cysteine (117), emphasizing how changes in amino acid and oxygen levels in the GI tract could alter the ability of *E. coli* to deplete azo dyes. These results emphasize the need to better model common nutritional and other environmental parameters that microorganisms experience in vivo.

The long-term clinical utility of these fundamental discoveries remains an open question. Multiple studies have established a proof of concept for the development of microbiome-based predictors of drug response (114, 118, 119). These efforts need to be replicated in larger cohorts and more directly compared with predictors built on host genotype, diet, or other more well-established risk factors. In parallel, it will be important to compare the relative effect sizes of these various factors under more controlled conditions using model organisms (e.g., mice, worms, zebrafish) or even in vitro. Now that we know the microbiome has broad impacts on pharmacology, the question is how the microbiome compares to and interacts with factors like diet and genetics that are known to influence pharmacokinetics/pharmacodynamics. It will also be essential to develop better strategies to engineer microbial metabolism to improve drug responses (120), for example, by using bacteriophage-delivered CRISPR-Cas systems for targeted gene delivery or removal (121) or the use of small-molecule inhibitors (54, 91). These efforts will enable the shift from simply using the microbiome as a diagnostic/prognostic factor to designing controlled microbiome-targeted interventions.

Finally, we hope that the studies reviewed herein emphasize that not only does the microbiome have a role in pharmacology, but that its role is far more complicated than previously appreciated. While the focus on the direct metabolism of drugs is reasonable given its long and well-established history, microorganisms can also influence other aspects of ADME and even drug pharmacodynamics. Microbial drug metabolism itself is also more complicated than previously appreciated, due to the dependence of these enzymes on environmental factors like diet and the potential for bioaccumulation of drugs and abiotic degradation. Dissecting all these layers of biology requires concerted efforts across many types of study systems and a renewed effort to train pharmacology students and pharmaceutical industry scientists in microbiology, genomics, and chemistry.

## Supplementary Material

SupplementalTAbles

## Abbreviations

Gut microbiota: the collection of microorganisms (archaea, bacteria, microscopic eukaryotes, and viruses) that inhabit the gastrointestinal tract
Gut microbiome: the aggregate genes and metabolic activities harbored by members of the gut microbiota
Xenobiotics: chemical substances foreign to the human body, including drugs and environmental compounds
Pharmacokinetics: the study of how the body processes drugs, typically focusing on absorption, distribution, metabolism, and elimination
Pharmacodynamics: the study of how drugs alter host physiology, typically focusing on drug mechanism of action
Biotransformation: modification of a chemical compound by an organism
Cardiac glycosides: a class of chemicals (including digoxin) used to treat heart failure by increasing heart muscle contractility
Comparative genomics: the study of similarities and differences between the genomes of different organisms (e.g., between different bacterial strains)
Transcriptomics: the study of which RNA transcripts are expressed under a given set of conditions
Germ-free (GF) mice: sterile mice housed in a microorganism-free environment
T helper 17 (Th17) cell: an IL-17-producing subtype of CD4^+^ T cells associated with autoimmunity
P-glycoprotein (P-gp): an ATP-binding cassette active transporter encoded by host gene *ABCB1* that pumps foreign substances out of cells
Statins: a class of medications used to lower blood cholesterol by inhibiting the cholesterol synthesis enzyme HMG-CoA reductase
Norepinephrine and epinephrine: compounds that act as both hormones and neurotransmitters involved in sympathetic nervous system activation (fight-or-flight response)
β-Glucuronidase: a class of enzymes involved in polysaccharide breakdown via hydrolysis
Irinotecan (CPT-11): a topoisomerase I inhibitor used to treat cancer by preventing DNA synthesis
5-Fluorouracil (5-FU): a nucleotide mimic used to treat cancer by inhibiting DNA synthesis and RNA processing
Gnotobiotic mice: germ-free or formerly germ-free mice colonized with a defined microbiota
Probiotics: live microorganisms delivered in food or other products intended to improve host health
Immune checkpoint inhibitor (ICI): a drug that enables T cell–mediated cancer killing by blocking repressive cancer cell–T cell interactions
Inosine: a purine nucleoside implicated in immunotherapy response
CAR-T cell: T cell with an extracellular engineered targeting domain and intracellular costimulation and activating domains; often used in the treatment of cancer
Levodopa (L-dopa): a compound used to treat Parkinson’s disease by being converted into dopamine in the brain
Serotonin transporter (SERT): a sodium-serotonin symporter that transports extracellular serotonin into the cell
Cytochrome P450: a family of enzymes involved in drug metabolism, most often via oxidation
Metagenomics: the study of genetic material isolated from all organisms (microbiota and host) present in a sample
